# Exploring the 3D Conformation of Hard‐Core Soft‐Shell Particles Adsorbed at a Fluid Interface

**DOI:** 10.1002/advs.202303404

**Published:** 2023-08-04

**Authors:** Jacopo Vialetto, Fabrizio Camerin, Shivaprakash N. Ramakrishna, Emanuela Zaccarelli, Lucio Isa

**Affiliations:** ^1^ Laboratory for Soft Materials and Interfaces Department of Materials ETH Zürich Vladimir‐Prelog‐Weg 5 Zürich 8093 Switzerland; ^2^ CNR Institute for Complex Systems Uos Sapienza P.le A. Moro 2 Roma 00185 Italy; ^3^ Department of Physics Sapienza University of Rome P.le A. Moro 2 Roma 00185 Italy; ^4^ Soft Condensed Matter & Biophysics Debye Institute for Nanomaterials Science Utrecht University Princetonplein 1 CC Utrecht 3584 The Netherlands; ^5^ Present address: Department of Chemistry & CSGI University of Florence, via della Lastruccia 3 Sesto Fiorentino Firenze I‐50019 Italy

**Keywords:** atomic force microscopy, colloidal particles, fluid interface, modeling, pNIPAM microgels

## Abstract

The encapsulation of a rigid core within a soft polymeric shell allows obtaining composite colloidal particles that retain functional properties, e.g., optical or mechanical. At the same time, it favors their adsorption at fluid interfaces with a tunable interaction potential to realize tailored two‐dimensional (2D) materials. Although they have already been employed for 2D assembly, the conformation of single particles, which is essential to define the monolayer properties, has been largely inferred via indirect or ex situ techniques. Here, by means of in situ atomic force microscopy experiments, the authors uncover the interfacial morphology of hard‐core soft‐shell microgels, integrating the data with numerical simulations to elucidate the role of the core properties, of the shell thicknesses, and that of the grafting density. They identify that the hard core can influence the conformation of the polymer shells. In particular, for the case of small shell thickness, low grafting density, or poor core affinity for water, the core protrudes more into the organic phase, and the authors observe a decrease in‐plane stretching of the network at the interface. By rationalizing their general wetting behavior, such composite particles can be designed to exhibit specific inter‐particle interactions of importance both for the stabilization of interfaces and for the fabrication of 2D materials with tailored functional properties.

## Graphical abstract

In situ atomic force microscopy and numerical simulations reveal new insights on the conformation of core‐shell microgels adsorbed at liquid–liquid interfaces. In particular, the protrusion of the core into the oil phase is only visible for thin shells, whereas thicker shells completely mask the core, leading to a conformation analogous to that of standard microgels.

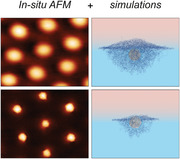

## 1 Introduction

The adsorption of colloidal particles at fluid interfaces is exploited in a variety of technological applications that make use of properties either originating at the single‐particle level (e.g., metal particles for plasmonics or as reaction catalysts)^[^ ^1^, ^2^, ^3^ ^]^ or that arise from their collective assembly (e.g., monolayers for interfacial stabilization or structural coloration).^[^ ^4^, ^5^ ^]^ Superior control on the adsorption and structural organization of colloidal monolayers is ensured by the use of soft particles, such as microgels. With respect to rigid charged colloids, microgels experience a decreased adsorption barrier to fluid interfaces, where they readily adsorb without the need of additives in suspension.^[^ ^3^, ^6^, ^7^ ^]^ Additionally, their soft nature can be exploited for a precise tuning of the interparticle potential within the interfacial plane, which can be governed either by varying the particle internal composition,^[^ ^8^ ^]^ or by compressing the fluid interface.^[^ ^9^, ^10^ ^]^ In this way, ordered structures with controlled spacing over a wide range of interparticle distances can be obtained, resulting in materials with tunable properties.^[^ ^9^, ^11^ ^]^ As a consequence, microgels are the focus of multiple recent studies that address either their wetting properties and interfacial conformation,^[^ ^8^, ^12^, ^13^, ^14^, ^15^, ^16^, ^17^ ^]^ or make use of their assemblies for fabricating functional materials.^[^ ^9^, ^18^ ^]^

The encapsulation of rigid colloids within a soft polymeric shell, i.e., core‐shell microgels, provides several advantages with respect to both “standard” microgels without a hard core and the parent rigid particles, combining functional features of hard colloids, like fluorescence or plasmon resonance, with a superior control over structural ordering and adsorption behavior as provided by the soft shell.^[^ ^19^, ^20^, ^21^, ^22^ ^]^ The interfacial organization of hard‐core soft‐shell systems can be advantageously tuned by varying the core‐shell ratio and the shell cross‐linking density in order to modulate the steric forces acting between particles and to obtain ordered structures with the cores positioned at precisely controlled distances.^[^ ^20^ ^]^ At the same time, matching predictions based on simple potentials, e.g., Jagla potentials, allows obtaining more complex structural assemblies as a function of monolayer compression, from hexagonal to chain or rhomboid phases.^[^ ^23^, ^24^ ^]^ Moreover, for interfacial stabilization, the soft shell allows a decrease of the energy barrier for adsorption typically found for hard particles, while the hard core provides rigidity to the monolayer and superior stability against coarsening.^[^ ^25^ ^]^ Notably, similar advantages have also been reported for rigid particles sterically stabilized with various polymer coatings, which have shown a remarkable ability to stabilize bubbles and drops compared to bare particles.^[^ ^26^, ^27^, ^28^ ^]^

The self‐assembly of core‐shell particles, and more generally of microgels at fluid interfaces has been extensively studied using the pendant drop technique, interfacial rheology, Langmuir troughs, static light scattering, and ex‐situ visualization of the colloids after transferring from the fluid interface onto solid supports.^[^ ^10^, ^24^, ^29^, ^30^, ^31^, ^32^ ^]^ Optical and confocal microscopy can instead provide insights into the dynamics of the adsorbed particles, as in the case of colloids with fluorescent or sufficiently large cores.^[^ ^33^, ^34^, ^35^ ^]^ However, such techniques do not provide sufficient information on the wetting properties and single‐particle interfacial conformation that are of fundamental importance in order to rationalize and predict their behavior. Up to date, details on their interfacial conformation have been obtained mainly by numerical simulations,^[^ ^36^ ^]^ free‐energy models,^[^ ^37^ ^]^ and cryo‐SEM experiments.^[^ ^10^, ^25^ ^]^

Conversely, current advances in atomic force microscopy (AFM) have allowed the in‐situ visualization of colloids adsorbed at fluid interfaces, providing novel insight into their structural organization and dynamics.^[^ ^38^, ^39^, ^40^, ^41^, ^42^ ^]^ When applied to microgels adsorbed at an oil‐water interface, AFM imaging enables the reconstruction of the complex 3D morphology of the polymer network.^[^ ^15^ ^]^ It can also be used to quantify the effect of different parameters, such as the solution temperature, polymer solubility in the organic phase and interfacial tension value, that modify the network morphology and have a significant effect on the resulting interparticle interactions.^[^ ^15^, ^43^ ^]^

In this work, we investigate the conformation of core‐shell microgels adsorbed at an oil‐water interface by exploiting in‐situ AFM for a precise quantification of both the microgels lateral dimension within the interfacial plane and of the protrusion of the polymer network into the organic phase, in order to assess their wetting properties and the influence of the internal rigid core as a function of the shell thickness. We then turn to numerical simulations to corroborate experimental results on the particle interfacial conformation and to extract complementary information such as the position of the internal core with respect to the interface. In addition, simulations are used to discuss the influence of the affinity of the core for the oil phase and that of the grafting density on the resulting interfacial behavior.

## 2 Results and Discussion

**Figure 1:**
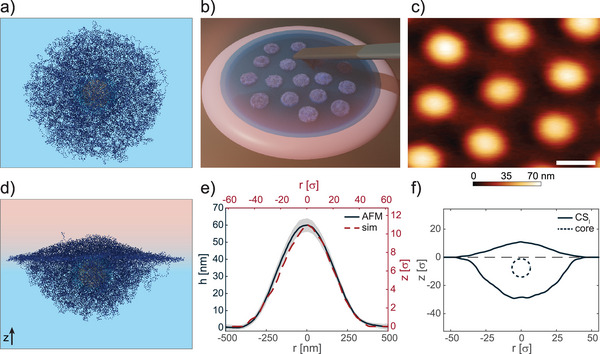
Core‐shell microgels at a fluid interface. a) Simulation snapshot of a core‐shell microgel in bulk water. For visual clarity, the size of shell and chains beads is smaller than that used in simulations. The microgel has a relatively large shell (*CS*_*l*_), similar to the *C*_*A*_ *S*_346_ experimental case. b) Sketch of the setup used for in situ AFM imaging of microgels adsorbed at the hexadecane‐water interface. c) AFM height image of a monolayer of *C*_*A*_ *S*_346_ captured from the oil side. Scale bar: 500 nm. d) Simulation snapshot of a *CS*_*l*_ core‐shell microgel after adsorption at an oil‐water interface. e) Black line: mean height profile of microgels as in c), the shaded region indicates the standard deviation of the height profiles averaged on the basis of at least ten particles. Red dashed line: simulated height profile of the polymer protrusion in the oil phase for *CS*_*l*_ microgel. f) Simulated core‐shell profile (solid line) and core position (dotted line) for *CS*_*l*_ microgel. Positive *z*‐values indicate the oil phase.

To quantify the wetting behavior and the interfacial conformation of hard‐core soft‐shell particles, as well as to understand the role played by the shell thickness, we experimentally investigated four types of microgels, characterized by two core sizes and four different core‐to‐shell ratios. The hard cores are made of poly(2,2,2‐Trifluoroethyl methacrylate) (pTFMA), which are then covered by a poly(N‐Isopropylacrylamide) (pNIPAM) shell with 5 mol % N,N'‐Methylenebis(acrylamide) (BIS) crosslinker. Details on the synthesis can be found in Materials, Models, and Methods. A visualization of a core‐shell microgel is provided by the simulation snapshot reported in **Figure** **1**a.

Dynamic light scattering is used to quantify the apparent hydrodynamic diameter (d_*h*_) as a function of temperature (Figure S1, Supporting Information) of the different microgels. We discriminate the various types of particles through their shell thickness at 21°C (when the pNIPAM network is swollen) and by the resulting core‐to‐shell ratio measured as d_*h*_ / d_*core*_, as indicated in **Table** **1**. In particular, *C*_*A*_ *S*_346_, *C*_*A*_ *S*_84,_ and *C*_*A*_ *S*_19_ have all the same core and a shell thickness of 346, 84, and 19 nm, respectively, as labeled in their name. The fourth particle, *C*_*B*_ *S*_101_, has the same hydrodynamic diameter as *C*_*A*_ *S*_84_, but a smaller internal core and consequently a larger shell thickness. The temperature response in bulk water (Figure S1, Supporting Information) is analogous for particles having shells above 80 nm, which are characterized by a similar deswelling profile as expected for shells composed of the same nominal amount of cross‐linker. Conversely, the shell of the *C*_*A*_ *S*_19_ microgel is so thin that the overall diameter only barely decreases with increasing temperature. However, the relative variation of the shell thickness is comparable to the other microgels (Figure S1, Supporting Information).

**Table 1:** Experimental particle dimensions

Microgel	d_*core*_ [nm]	d_*h*_ (21°C) [nm]	Shell thickness [nm]	d_*h*_/d_*core*_
*C*_*A*_ *S*_346_	140.4 ± 0.6	832 ± 17	346	5.93
*C*_*A*_ *S*_84_	140.4 ± 0.6	308 ± 3	84	2.19
*C*_*A*_ *S*_19_	140.4 ± 0.6	178 ± 2	19	1.27
*C*_*B*_ *S*_101_	102.3 ± 0.6	304 ± 4	101	2.97

We investigated the conformation of our core‐shell microgels adsorbed at an oil‐water interface with in‐situ AFM imaging in order to quantify their interfacial deformation, protrusion height, and polymer reorganization upon adsorption. To this aim, we recorded topographical images (see Materials, Models, and Methods) at 25°C with the tip immersed in the oil phase (see sketch in Figure 1b), therefore capturing the polymer network exposed to the oil side. A typical AFM height image of a monolayer of *C*_*A*_ *S*_346_ microgels adsorbed at the hexadecane‐water interface is reported in Figure 1c, while the resulting particle height profile is shown in Figure 1e (black curve). The pNIPAM network is stretched on the interfacial plane and it protrudes into the oil phase up to a maximum height of 60 ± 4 nm, with a continuous decrease of the polymer content up to the visible particle periphery. The resulting interfacial diameter is *d*_*int*_ = 872 ± 18 nm; a similar value could be obtained either by fitting the microgel profiles resulting from the adhesion contrast between the tip and the sample with a circle (Figure S2, Supporting Information), or by measuring the center‐to‐center distance. We remark that *d*_*int*_ might be slightly underestimated as the microgels are in contact and the outer loosely cross‐linked pNIPAM chains could be compressed by their neighbors.

For such microgels, we note that the hard core appears to be completely buried within the polymer network and its location is not discernible. Insights on the precise position of the buried hard core can be gained by studying a microgel with a similar core‐to‐shell size ratio *CS*_*l*_ (*l* standing for large shell thickness) by means of numerical simulations. To this aim, the in silico core‐shell microgel is assembled by linking a disordered polymer network onto a rigid core. To have a rough control over the grafting density, we also attach short polymer chains to the core (see Figure 1a and Figure S3, Supporting Information). Contrary to the polymer network, the core is assigned a stronger affinity for the apolar phase, for better matching the hydrophobic character of the pTFMA core. Simulations are then carried out in an explicit solvent in order to reproduce surface tension effects between oil and water. The conformation of the *CS*_*l*_ microgel when adsorbed at a fluid interface is shown in Figure 1d. Further details on the numerical microgel synthesis, simulation methods, and parameters are reported in Materials, Models, and Methods.

An excellent agreement between the profile of the simulated and experimental core‐shell microgel captured from the oil side can be evidenced in Figure 1e. At the same time, simulations also give information onto the particle protrusion into the water phase, and on the position of the internal hard core.

The microgel profile reported in Figure 1f clearly shows that most of the polymer network protrudes into water due to the higher affinity of the polymer for such phase. Interestingly, despite the higher affinity of the core for the oil, this is effectively shielded by the soft shell that keeps it entirely within the water phase. Therefore, consistently with experiments, the overall profile for such a large shell thickness resembles that of a “standard” (with no hard core) microgel and with similar cross‐linking density.^[^ ^15^ ^]^ Importantly, under these conditions, the core does not influence in a significant manner the 3D conformation of the adsorbed particle. The results reported here corroborate also calculations by Vasudevan et al.,^[^ ^37^ ^]^ which showed that an increase of the overall particle diameter (at fixed core size) beyond a critical value results in a final conformation where the hard core remains fully immersed into the water phase. As a result, for sufficiently thick shells the core does not hinder the spreading of the polymer network on the interfacial plane, and the equilibrium conformation is reached for full in‐plane stretching and limited amount of polymer in the organic phase.

**Figure 2:**
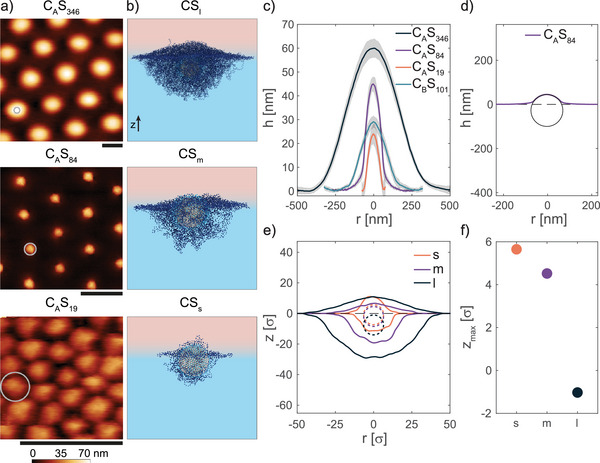
Influence of the shell thickness on the conformation of core‐shell microgels at a fluid interface. a) AFM height images of microgel monolayers captured from the hexadecane side. The gray circle represents the core size. Scale bars: 500 nm. b) Simulation snapshots of core‐shell microgels adsorbed at an oil‐water interface for different shell thicknesses. c) Mean height profiles of microgels as in a). The corresponding AFM image for *C*_*B*_ *S*_101_ is reported in Figure S4 (Supporting Information). The shaded regions indicate the standard deviation of the height profiles calculated on at least ten particles. d) Fit with a circle of the central portion of the height profile of *C*_*A*_ *S*_84_ microgel. The fitted radius is R = 72.3 nm. e) Height profiles of in silico core‐shell microgels (solid line) and core position (dotted line). Positive *z‐*values indicate the oil phase. f) Protrusion height of the core with respect to the interfacial plane corresponding to z = 0 for the three in silico core‐shell microgels.

Next, we investigated the particle's interfacial conformation as a function of shell thickness (**Figure** **2**). In Figure 2a we report a series of AFM height images captured from the hexadecane side of microgels with the same core and decreasing core‐to‐shell ratio. The corresponding height profiles are reported in Figure 2c. Decreasing the shell thickness from *C*_*A*_ *S*_346_ to *C*_*A*_ *S*_84_ results in a qualitatively different 3D conformation of the adsorbed particle. In particular, we detect a protrusion of the hard core into the oil phase, as evidenced by a fitting of the central portion of the profile with a circle (Figure 2d), which gives a radius of 72.3 nm, consistent with our hard core dimension. We note that the position of the circle in Figure 2d does not reflect the actual core position with respect to the interfacial plane due to the presence of a non‐negligible amount of collapsed pNIPAM polymer above the hard core, the thickness of which is hard to discern from the AFM height images.

The surrounding pNIPAM network is still significantly stretched on the interfacial plane, reaching an interfacial diameter *d*_*int*_ = 434 ± 11 nm. As a consequence, the hard cores of the adsorbed particles are separated at distances much greater than their diameters, and the interparticle interactions are mediated by the contacts between the stretched soft shells. These results qualitatively confirm previous findings showing that the structural and mechanical properties of CS microgels with stretched out pNIPAM shells are mainly governed by the shell compliance in the regime of low to intermediate surface pressures, in which the polymer network is still deformable.^[^ ^10^, ^24^ ^]^ However, they also show that the wetting properties markedly depart from that of CS microgels with thicker shells (which behave similarly to “standard” ones, Figure 1), since the protrusion height into the oil phase is significantly increased.

This effect is clearly visible when comparing CS particles having a smaller hard core size but similar bulk dimensions (*C*_*B*_ *S*_101_ microgels in Figure S4, Supporting Information and corresponding turquoise profile in Figure 2c), and it allows to assess a critical thickness value of the pNIPAM shell above which the core does not influence in a visible way the microgel interfacial conformation. Despite having the same d_*h*_ and cross‐linker content, the maximum particle height decreases from 45 ± 3 nm to 29.1 ± 2.6 nm, while *d*_*int*_ increases from 434 ± 11 nm to 488 ± 17 nm, for *C*_*A*_ *S*_84_ and *C*_*B*_ *S*_101_, respectively (Figure 2c). Additionally, the overall shape of the height profile of *C*_*B*_ *S*_101_ is similar to that of the *C*_*A*_ *S*_346_ microgel, with the hard core buried within the pNIPAM network and not visible in the AFM height images (Figure S4, Supporting Information). This clearly indicates that the hard core has an effect on the wetting properties of the particles when the shell thickness is lower than a given threshold, which is of about 100 nm for the particles investigated here. Interestingly, also calculations based on linear elasticity theory on core‐shell spheres subject to an equatorial tensile force have shown that above a certain shell thickness, the deformation of the soft shell becomes independent of the elastic properties of the core.^[^ ^44^ ^]^ While this has limited effects on the structural organization of CS microgel monolayers as a function of surface pressure, it might have profound implications in foams/emulsions stabilization.^[^ ^45^, ^46^ ^]^

Finally, we imaged monolayers of CS microgels with a very thin polymer shell (*C*_*A*_ *S*_19_, Figure 2c). Interestingly, such particle has $d_{h}/d_{\mathrm{core}}=1.27$ that is well below the lower range reported in literature investigating core‐shell microgels at fluid interfaces,^[^ ^10^, ^25^, ^36^, ^37^ ^]^ which is typically around 1.7, and the shell thickness resembles that of rigid particles stabilized with polymeric coatings.^[^ ^26^, ^27^ ^]^ Despite the very thin soft shell, also these particles readily adsorb at the oil‐water interface (Figure S5, Supporting Information). However, the height profile on the oil side resembles that of rigid particles without a soft shell; indeed, almost the entire profile can be fitted with a circle (see Figure S6, Supporting Information, R = 74.1 nm), leaving only a very small region in the particle periphery that departs from a circular shape. Fitting of the profiles clearly indicates that the adsorbed particles are in contact in the bulk water phase, i.e., below the interface. A consequence of this is that the interparticle interactions in such monolayers are presumably mediated by soft contacts in bulk water between the swollen pNIPAM networks, and not by contacts on the interfacial plane, as is the case for microgels having thicker shells.

In simulations, we analyze the behavior of two additional core‐shell microgels with a progressively decreasing shell thickness, namely *CS*_*m*_ and *CS*_*s*_, with *m* and *s* indicating a medium and a small shell thickness with respect to the ‘large’ one introduced earlier. In particular, *CS*_*s*_ roughly represents the smallest possible core‐shell particle that can be assembled in silico for the chosen core size, having a clearly identifiable shell around the core (see also Materials, Models, and Methods). Simulation snapshots of representative configurations are reported in Figure 2b. The corresponding height profiles and locations of the core are shown in Figure 2e. The trend observed in simulations is in qualitative agreement with the experiments. In particular, both *CS*_*m*_ and *CS*_*s*_ show a conformation in which the hard core protrudes into the oil phase. The extent of such protrusion increases upon decreasing the soft shell thickness, as evidenced by plotting the protrusion height of the core with respect to the interface plane (*z*_*max*_ in Figure 2f). Notably, for the microgels with the thinnest shell, the core is essentially located half in the water and half in the oil side, “dragging” the shell into the oil phase, despite it being a bad solvent for the pNIPAM network. Overall, these results indicate that the thickness of the shell plays an important role not only in tuning the hard core position with respect to the interfacial plane, but also in determining the final conformation of the polymer network in the two phases.

**Figure 3:**
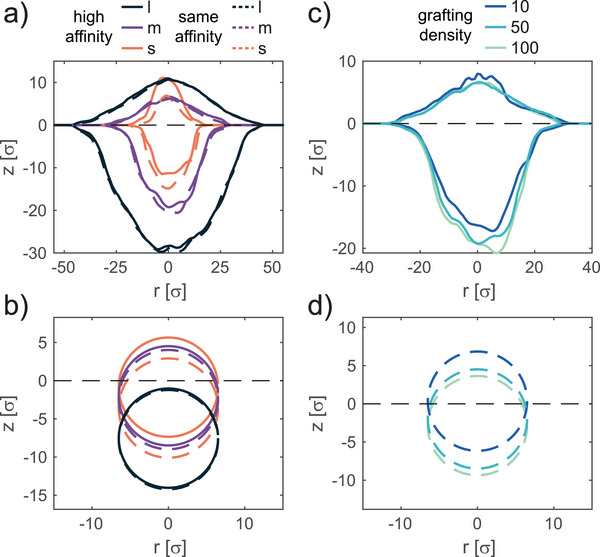
Influence of core affinity to the oil phase and of the grafting density on the conformation of adsorbed core‐shell microgels. a) Height profiles and b) location of the core for in silico core‐shell microgels with a core that retains a higher affinity to the oil phase as compared to the polymer chains (full lines), and with a core that retains the same affinity as the polymer chains for water and oil phases (dashed lines), for *CS*_*s*, *m*, *l*_. c) Height profiles and d) location of the core for microgels with varying grafting density $CS_{m}^{10}$, *CS*_*m*_, and $CS_{m}^{100}$. Data are only for microgels whose core has a higher affinity to the oil phase. In all cases, negative *z*‐values are for the water phase.

It is also interesting to study the conformation that the core‐shell microgel would retain if the core had a different affinity to the oil phase or for varying grafting densities. These conditions can easily be explored in simulations, either by adjusting the core‐solvent interactions parameters or by tuning the amount of added polymer chains linked to the core. The outcomes of these additional investigations are reported in **Figure** **3**(a,c) for the overall core‐shell microgel and in panels (b,d) for the core only.

We start by discussing the case of a microgel in which the core has the same affinity as the polymeric shell for the two phases. In this case, the position of the microgel is shifted toward water with respect to the cases described earlier in which the core had more favorable interaction with the apolar phase. The effect is more pronounced for the microgel with the thinnest shell whereas there are only minimal differences for the one with the thickest shell. Looking at the profiles of the core, we can observe how the shift is entirely attributable to the core itself. This is indeed reasonable to expect, since a thin shell allows for a greater adjustment of the microgel position as opposed to a particle with a thicker shell that provides a greater adhesion to the plane of the interface.

A similar effect can be obtained by adjusting the grafting density of the polymer network to the core. While experimentally this parameter is typically difficult to control, in simulations, we can tune the number of short polymer chains directly linked to the core. Specifically, besides the *CS*_*m*_ microgel, we previously analyzed, in which half of the core surface was coated by polymer chains, we consider a core‐shell microgel in which only 10% of the core surface is covered, $CS_{m}^{10}$, and one in which all the core beads are linked to a polymer chain, $CS_{m}^{100}$ (Figure S7, Supporting Information). In all cases, the core has a greater affinity to the oil phase. By increasing the coverage of the core, we find that the overall affinity of the microgel to the apolar phase diminishes causing a shift toward water. The greatest effect is observed by moving from the 10% to the 50% coverage, while above this threshold only minor effects take place. Therefore, microgels whose core is highly exposed to the water phase could be obtained by either synthesizing a thick shell or by linking to the core a dense polymer network for which we can expect the grafting density to be high.

## 3 Conclusion

In this work, we have shown how AFM imaging at an oil‐water interface can provide fundamental novel insights on the 3D conformation of adsorbed hard‐core soft‐shell particles. The coupling with numerical simulations allowed us to corroborate the experimental data and to gain additional information not accessible from AFM imaging, such as the position of the buried hard core with respect to the interface plane. Overall, these findings evidence a clear link between the shell thickness, the particle position at the interface plane, and the deformation of the soft network, opening the way to new opportunities for applications such as smart interface stabilizers^[^ ^18^, ^25^ ^]^ or for the design of complex architectures.^[^ ^23^, ^47^ ^]^

In particular, the results demonstrate that significant variations in the particle wetting properties with respect to standard microgels can be obtained for d_*h*_ / d_*core*_ ⩽ 2.2 (corresponding to *C*_*A*_ *S*_84_ and *C*_*A*_ *S*_19_ microgels). For such particle characteristics, the shell deformation on the interface plane decreases and the core protrudes out in the oil phase transferring an increased amount of collapsed polymer in the organic solvent. Instead, for higher core‐to‐shell ratios, an increased spreading of the soft network is observed, limiting the protrusion of the polymer in the organic phase, and maintaining the core buried within the aqueous phase, similarly to what has been observed with FreSCa cryo‐SEM.^[^ ^10^ ^]^ All results presented here refer to a cross‐linker content in the shell of 5 mol. %. Variations in the cross‐linking density are expected to strongly affect the overall position of the core‐shell microgels. In the limit of very loosely cross‐linked, or compliant, shells we expect the effect of the core to be amplified and more polymer chains to partake in the adsorption at the interface, while for highly cross‐linked, or stiff, shells the presence of the core will be progressively masked. Overall, we believe that our findings might provide guidelines to further tune the wetting properties of hard‐core soft‐shell systems, in turn affecting the inter‐particle interactions at the interface, the mechanical stability of the resulting monolayers, and the attained structures.

## 4 Experimental Section

### Reagents

N‐Isopropylacrylamide (NIPAM, TCI 98.0%) was purified by recrystallization in 40/60 v/v toluene/hexane. *N*,*N*'‐Methylenebis(acrylamide) (BIS, Fluka 99.0%), 2,2,2‐trifluoroethyl methacrylate (TFMA, Sigma–Aldrich 99.0%) potassium persulfate (KPS, Sigma–Aldrich 99.0%), nile red (Sigma–Aldrich, for microscopy grade), sodium dodecyl sulfate (SDS, Sigma–Aldrich 99.0%) isopropanol (Fisher Chemical, 99.97%), toluene (Fluka Analytical, 99.7%), n‐hexadecane (Acros Organics 99.0%), and n‐hexane (Sigma–Aldrich, HPLC grade 95%) were used without further purification.

### Microgels Synthesis

Core‐shell microgels were synthesized in a two‐step process.

*Hard core*. pTFMA‐cores were synthesized using free radical emulsion polymerization. TFMA (10 mL) and nile red (3 mg) were added to an aqueous solution (30 mL) of NIPAM (940 mg) and SDS. The amount of SDS was varied in order to control the final particle size. In particular, we added 60.66 mg and 20.96 mg of SDS for cores pTFMA‐B and pTFMA‐A, respectively. The reaction mixture was heated up to 70°C, stirred at 600 rpm and purged with nitrogen for 1 h. KPS (25 mg), previously dissolved in water (2,5 mL) and purged with nitrogen, was added to the mixture to start the reaction. The reaction was carried out for 4 h. Afterward, the resulting particles were filtered and purified by dialysis for one week (membrane: 12 ‐ 14 kDa cut‐off), and by four centrifugation cycles and resuspension in pure water. Hydrodynamic diameters (d_*core*_) measured by DLS: pTFMA‐B 102.3 ± 0.6 nm; pTFMA‐A 140.4 ± 0.6 nm.

*Soft shell*. The soft pNIPAM shell was synthesized by free radical precipitation polymerization in the presence of the hard cores as seeds. NIPAM (see **Table** **2** for quantities) and 5 mol % BIS were dissolved in 50 mL of Milli‐Q water at room temperature and purged with nitrogen for 1 h. Afterward, 40 mL of the monomer solution was taken out with a syringe. 10 mL of pTFMA cores in Milli‐Q water (see Table 2 for quantities) were added to the reaction flask and the solution was immersed into an oil bath at 80°C and purged with nitrogen for 1 h. Varying the amount of NIPAM and cores in the reaction mixture allowed to tune the core‐shell ratio for the different particles. The reaction was initiated by adding 0.5 mol % of KPS previously dissolved in 2 mL Milli‐Q water and purged with nitrogen. After 2 min, feeding of the monomer solution (0.5 mL· *min* ^−1^) into the reaction flask was started. When the feeding was terminated, the reaction was quenched by opening the flask in air and placing it in an ice bath. The obtained colloidal suspension was cleaned by dialysis for one week (membrane: 12 – 14 kDa cut‐off), and by eight centrifugation cycles and resuspension in pure water. A polymerization reaction by continuous monomer addition was chosen over the more common batch reaction in order to ensure a more homogeneous shell growth.^[^ ^48^, ^49^ ^]^

**Table 2:** Amount of monomers and cores used to tune the core‐shell ratio

Microgel	Core	Core particle suspension [mL]	NIPAM [g]	d_*h*_ (21°C) [nm]	d_*h*_/d_*core*_
*C*_*B*_ *S*_101_	pTFMA‐B	2	0.5	304 ± 4	2.97
*C*_*A*_ *S*_84_	pTFMA‐A	8	0.5	308 ± 3	2.19
*C*_*A*_ *S*_19_	pTFMA‐A	8	0.25	178 ± 2	1.27
*C*_*A*_ *S*_346_	pTFMA‐A	1	0.4	832 ± 17	5.93

### Experimental Methods

*DLS*. Dynamic light scattering (DLS) experiments were performed using a Zetasizer (Malvern, UK). The temperature was varied from 19 to 51°C with 2°C steps. At each temperature, the sample was let to equilibrate for 15 min before performing four consecutive measurements.

*AFM Imaging and Analysis*. Imaging of microgels in situ at the oil‐water interface was carried out by using a Bruker Dimension Icon AFM, following a procedure already reported elsewhere.^[^ ^15^ ^]^ A small well on piece of silicon wafer (Si‐Mat, Landsberg, Germany) was made by applying a drop of UV curable glue (Norland Optical Adhesive 81). This well (average depth of 2 – 10 µm) acts as a reservoir for containing the subphase (water). The silicon wafer was then glued to a bio heater cell (MFP 3D, Asylum research, Oxford instrument). Before each experiment, the cell was cleaned with ethanol and the silicon wafer was plasma cleaned for 10 s using a plasma pen (Piezobrush®PZ2, Reylon Plasma GMBH, Germany). The well was then filled with approximately 5 µL of the microgel suspension in water. After 5 min, the entire cell was covered with oil (hexadecane), the cantilever was immersed in the oil phase and the AFM laser was turned on. A minimum waiting time of 1 h was required to let the cell's temperature to equilibrate, and for the cessation of residual convection after fluid injection.

AFM imaging at the fluid interface was carried out by using PeakForce tapping mode with cantilevers with a nominal spring constant of ≈0.12*N* · *m* ^−1^ (PEAKFORCE‐HIRS‐F‐B, Bruker). The tip was approached to the interface by setting a PeakForce set point of 100 pN, and adjusted slightly along with the feedback gains after engaging the interface. The PeakForce during imaging was varied in the 100 – 500 pN range with the aim of obtaining height images with the highest quality. The PeakForce amplitude was varied between 100 – 300 nm. The oscillation frequency was chosen between 1 – 2 KHz, and the scanning speed between 0.2 – 1 Hz.

All AFM images were first processed with open‐source software Gwyddion and successively analyzed with custom MATLAB codes. In order to obtain an averaged height profile, for each microgel, horizontal and vertical profiles passing through its center were extracted. Subsequently, an average over at least ten microgels was obtained by aligning each profile by its center value.

*Interfacial tension*. The evolution of the interfacial tension value of a suspension of microgels (0.5 wt.%) in water as a function of time was measured using a Drop Shape Analyzer system (KRÜSS DSA100E) analyzing the shape of a water drop immersed in hexadecane.

### Numerical Methods

*In silico assembly of core‐shell (CS) microgels*. We assembled core‐shell (CS) microgels by linking a disordered polymer network to a rigid core. The latter consists of a sphere that was uniformly tessellated with *N*_*core*_ = 642 monomers of size σ, which was also taken as the unit of length in simulations. The core was treated as a rigid body and its overall diameter was 13σ. Then, the core surface was randomly covered with a varying number of very short polymer chains *N*_*chains*_ of length $N_{chains}^{length}$, that was slightly adjusted to the thickness of the shell. The disordered network constituting the shell of the microgel was assembled starting from *N*_*shell*_ patchy particles with two and four patches, which were aimed at mimicking the polymer monomers and the cross‐linkers, respectively. We let the assembly occur in a spherical shell of thickness δ = *Z*_*out*_ − *Z*_*in*_, with *Z*_*in*_ the inner radius and *Z*_*out*_ the outer radius, until > 99.9% of all possible bonds were formed. The same procedure was also exploited in Refs. [8, 50] for the assembly of hollow microgel particles. Subsequently, the core with the grafted polymer chains were inserted in the cavity of the hollow polymer network and bonds were created between chains and polymer network, thus forming a core‐shell microgel. The use of the short polymer chains allowed to achieve a better control of the grafting density of the polymer network onto the rigid core. Starting from the smallest possible particle for which a shell was clearly identifiable without its number of monomers being excessively small, we assembled microgels with increasing shell thickness and varying grafting density, whose parameters are summarized in **Table** **3**.

**Table 3:** Structural features and parameters for the assembly of in silico core‐shell (CS) microgels. The subscripts *l*, *m* and *s* stand for *large*, *medium* and *small* respectively, with reference to the thickness of the shell, while the superscripts 10 and 100 refer to the coverage percentage of the core with the added chains

Microgel	*N*_*chains*_	$N_{chains}^{length}$	% coverage	*N*_*shell*_	δ[σ]
CS_*l*_	321	5	50	25489	36
CS${}_{m}^{10}$	64	3	10	5745	15
CS_*m*_	321	3	50	5745	15
CS${}_{m}^{100}$	642	3	100	5745	15
CS_*s*_	321	2	50	1429	10

*Interaction potentials and simulations*. For a core‐shell microgel, interactions occur according to the Kremer‐Grest bead‐spring model, where all monomers experience a Weeks‐Chandler‐Anderson (*V*_WCA_) potential where ϵ sets the energy scale and *r* was the distance between two monomers. In addition, connected beads also interact via the Finitely Extensible Nonlinear Elastic (FENE) potential, with *k*_*F*_ = 15 determining the stiffness of the bond and *R*_0_ = 1.5 the maximum bond distance. Interactions between monomers of the core were not taken into account.

To study the conformation of the core‐shell microgels at the water‐oil interface, we carried out simulations in the presence of explicit solvent particles. In this way, we accounted for the effect of the surface tension between the two solvents. Solvent was modeled within the Dissipative Particle Dynamics (DPD) framework.^[^ ^51^ ^]^ The interaction force among solvent beads is ${\vec{F}}_{ij}={\vec{F}}_{ij}^{C}+{\vec{F}}_{ij}^{D}+{\vec{F}}_{ij}^{R}$, where:

(1) $${\vec{F}}_{ij}^{C}=a_{ij}w\left(r_{ij}\right){\hat{r}}_{ij}$$

(2) $${\vec{F}}_{ij}^{D}=-\gamma w^{2}\left(r_{ij}\right)\left({\vec{v}}_{ij}\cdot {\vec{r}}_{ij}\right){\hat{r}}_{ij}$$

(3) $${\vec{F}}_{ij}^{R}=2\gamma \frac{k_{B}T}{m}w\left(r_{ij}\right)\frac{\theta }{\sqrt{\Delta t}}{\hat{r}}_{ij}$$

where ${\vec{F}}_{ij}^{C}$ is a conservative repulsive force, with *w*(*r*_*ij*_) = 1 − *r*_*ij*_/*r*_*c*_ for *r*_*ij*_ < *r*_*c*_ and 0 elsewhere, ${\vec{F}}_{ij}^{D}$ and ${\vec{F}}_{ij}^{R}$ are a dissipative and a random contribution of the DPD, respectively; *a*_*ij*_ quantifies the repulsion between two particles, γ = 2.0 is a friction coefficient, θ is a Gaussian random variable with zero average and unit variance, and Δ*t* = 0.002 is the integration time‐step. The cut‐off radius is set to be *r*_*c*_ = 1.9σ and the reduced solvent density ρ_DPD_ = 4.5. For the entire system, we fixed the reduced temperature T* = 1 via the DPD thermostat. Following previous works,^[^ ^8^, ^12^, ^52^ ^]^ we chose *a*_ww_ = *a*_oo_ = 8.8, *a*_wo_ = 31.1 in order to reproduce a standard water/oil (w/o) interface. Similarly, for the monomers belonging to the microgel polymer network or to the added short chains, we set *a*_mw_ = 4.5 and *a*_mo_ = 5.0. Instead, core‐solvent interactions were varied to study the effect of a different core affinity for the oil phase on the overall core‐shell microgel conformation. For this reason, *a*_cw_ and *a*_co_ were either set as *a*_mw_ and *a*_mo_ for a core having the same affinity of the polymer chains and network, or *a*_cw_ = 5.0 and *a*_co_ = 1.0 for a core having an enhanced affinity for the oil phase. Considering the size of the biggest microgel studied *CS*_*l*_ and its increased extension when adsorbed at the interface, the number of solvent particles required to perform such simulations exceeds 3.1 × 10^6^. Simulations were performed with the lammps simulation package.^[^ ^53^ ^]^

*Analysis*. To best reproduce the experimental technique for studying the conformation of the core‐shell microgels, numerically, we calculated the average maximum profile in the *z* direction in both oil and water sides. Specifically, we created a grid in the (*x*, *y*) interfacial plane and for each of the quadrants, we took the position of the monomer with the highest and lowest value in *z* in the water and oil side, respectively. This was then averaged over multiple snapshots of the equilibrated simulation runs. The positions of the core were simply determined by averaging the position of their center of mass.

## Conflict of Interest

The authors declare no conflict of interest.

## Author Contributions

Author contributions are defined based on the CRediT (Contributor Roles Taxonomy) and listed alphabetically. Conceptualization was performed by L.I., J.V.; Formal analysis was performed by F.C., J.V. ; Funding acquisition was performed by L.I., J.V., and E.Z.; Investigation was done by F.C., L.I., S.N.R., J.V., and E.Z.; Methodology was performed by F.C., S.N.R., J.V., and E.Z.; Project administration was performed by L.I. ; Software was performed by F.C. ; Supervision was performed by L.I. and E.Z.; Validation was done by F.C., S.N.R., and J.V. ; Visualization was performed by F.C., L.I., and J.V.; Writing ‐ original draft was done by F.C., L.I., and J.V.; Writing ‐ review and editing was done by F.C., L.I., J.V., and E.Z.

## Supporting information

Supporting Information

## Data Availability

The data that support the findings of this study are available from the corresponding author upon reasonable request.
