# Supplementary material for: Exploring the 3D Conformation of Hard‐Core Soft‐Shell Particles Adsorbed at a Fluid Interface
Source: Adv Sci (Weinh). 2023 Aug 4;10(28):2303404. doi: 10.1002/advs.202303404 (PMC10558683; doi:10.1002/advs.202303404)
Supplement: Supplementary file 1 — Supporting Information [file ADVS-10-2303404-s001.pdf]

ADVANCED SCIENCE
Open Access

Supporting Information

for *Adv. Sci.*, DOI 10.1002/advs.202303404

Exploring the 3D Conformation of Hard-Core Soft-Shell Particles Adsorbed at a Fluid Interface

Jacopo Vialetto, Fabrizio Camerin*, Shivaprakash N. Ramakrishna, Emanuela Zaccarelli* and Lucio Isa**

Exploring the 3D conformation of hard-core soft-shell particles adsorbed at a fluid interface

Supporting Information

Jacopo Vialetto[a],[*,†] Fabrizio Camerin,[*,‡,¶,§] Shivaprakash N. Ramakrishna,[†]

Emanuela Zaccarelli,[*,‡,¶] and Lucio Isa[*,†]

†*Laboratory for Soft Materials and Interfaces, Department of Materials, ETH Zürich, Vladimir-Prelog-Weg 5, 8093 Zürich, Switzerland*

‡*CNR Institute for Complex Systems, Uos Sapienza, P.le A. Moro 2, 00185 Roma, Italy*

¶*Department of Physics, Sapienza University of Rome, P.le A. Moro 2, 00185 Roma, Italy*

§*Soft Condensed Matter & Biophysics, Debye Institute for Nanomaterials Science, Utrecht University, Princetonplein 1, 3584 CC Utrecht, The Netherlands*

E-mail: jacopo.vialetto@unifi.it; f.camerin@uu.nl; emanuela.zaccarelli@cnr.it; lucio.isa@mat.ethz.ch

[a] Current address: Department of Chemistry & CSGI, University of Florence, via della Lastruccia 3, Sesto Fiorentino, I-50019 Firenze, Italy.

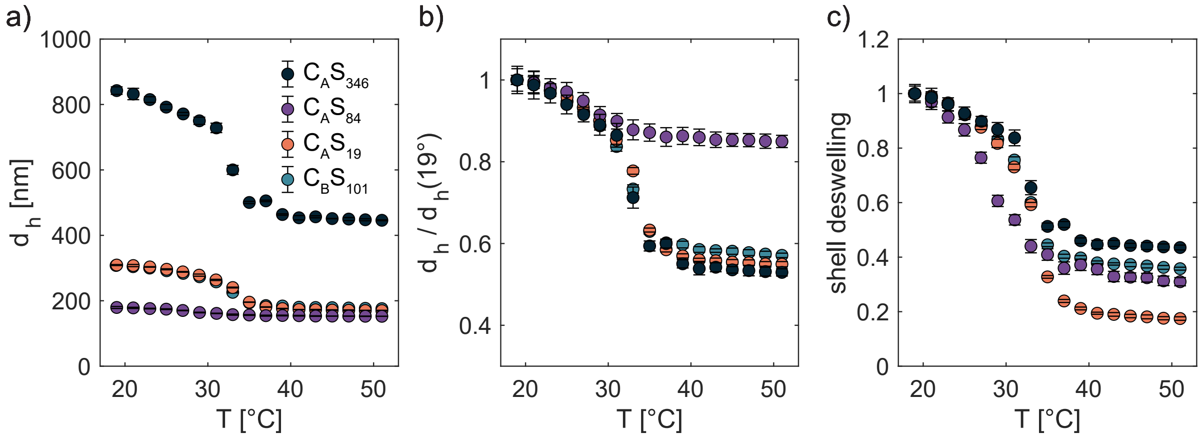

Figure S1: **Microgels' hydrodynamic diameters as a function of temperature.** a) Experimental hydrodynamic diameter d_h measured by DLS for the investigated microgels. Error bars indicate the standard deviation of 4 measurements consisting of 13 runs each. b) Deswelling profile as a function of temperature measured as $d = d_h/d_h(19°C)$. Shell deswelling as a function of temperature.

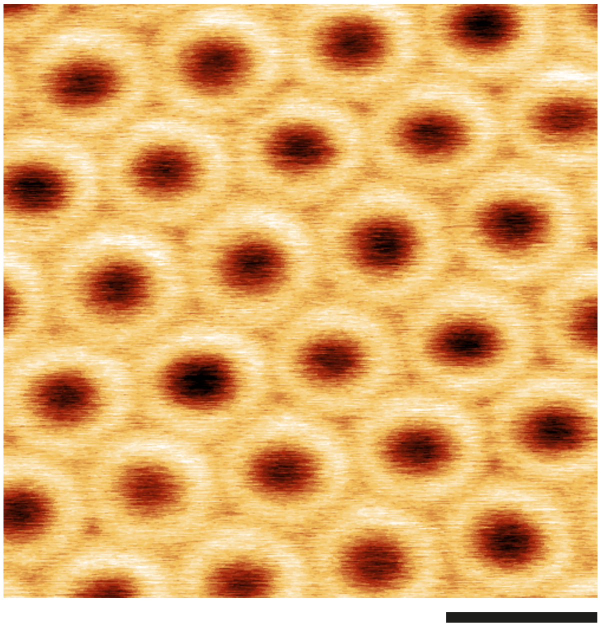

Figure S2: **AFM adhesion image at the hexadecane-water interface.** AFM adhesion image captured from the oil side for C_AS_{346} microgels. Scale bar: 1 μm.

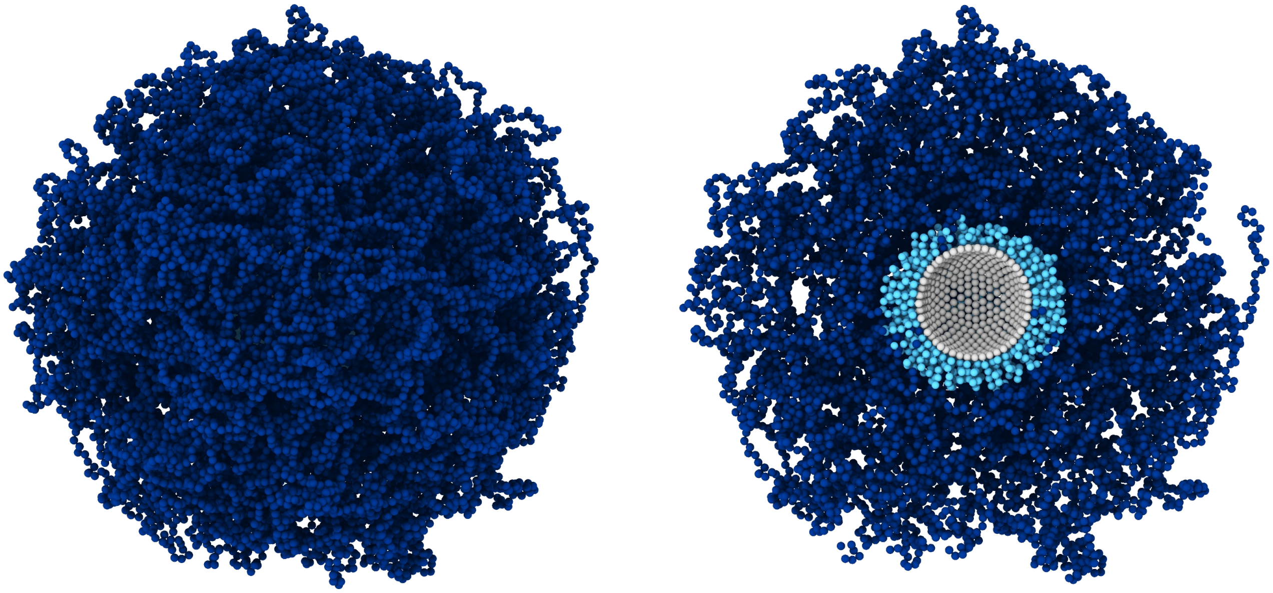

Figure S3: (Left) Full and (right) cross-section simulation snapshots showing the CS_l core-shell microgel in bulk. Blue beads belong to the shell of the microgel, light blue is for the added polymer chains through which the grafting density is tuned, and grey is for core beads.

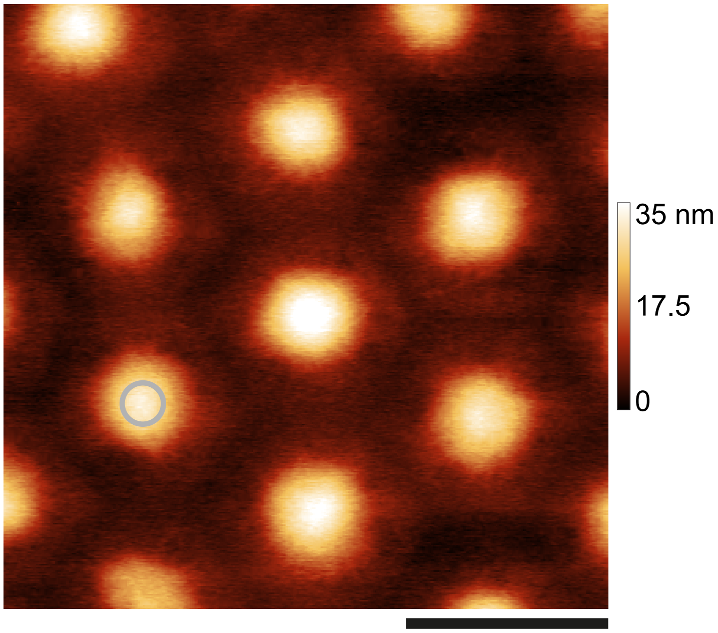

Figure S4: **AFM height image of a monolayer of C_BS_{101} microgels.** AFM height image at the oil-water interface captured from the hexadecane side. The gray circle represents the core size. Scale bar: 500 nm.

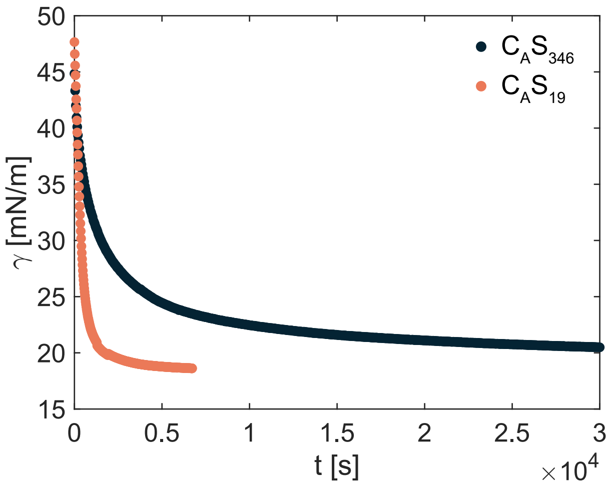

Figure S5: **Interfacial tension of microgel suspensions.** Interfacial tension as a function of time for a pendant drop of microgel suspension in water immersed in hexadecane. Microgel concentration: 0.5 wt %.

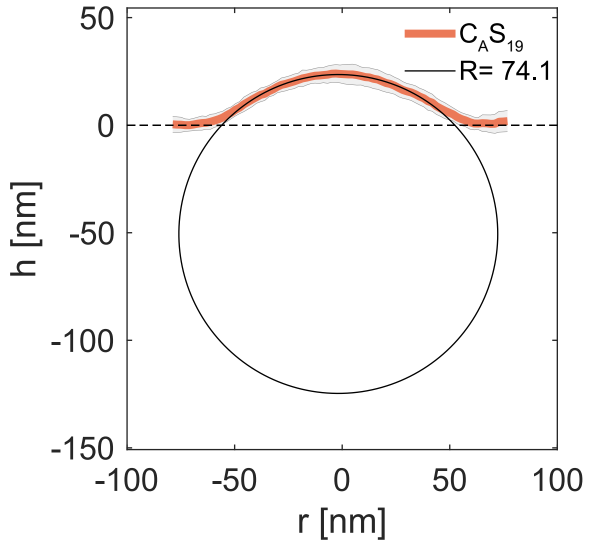

Figure S6: **Fit of the AFM height profile of adsorbed $C_A S_{19}$ microgels.** The experimental height profile (orange line) calculated from the AFM images at the hexadecane-water interface is the same as the one reported in Figure 2. The central portion of the profiles is fitted with a circle (balck line) with a resulting radius R = 74.1.

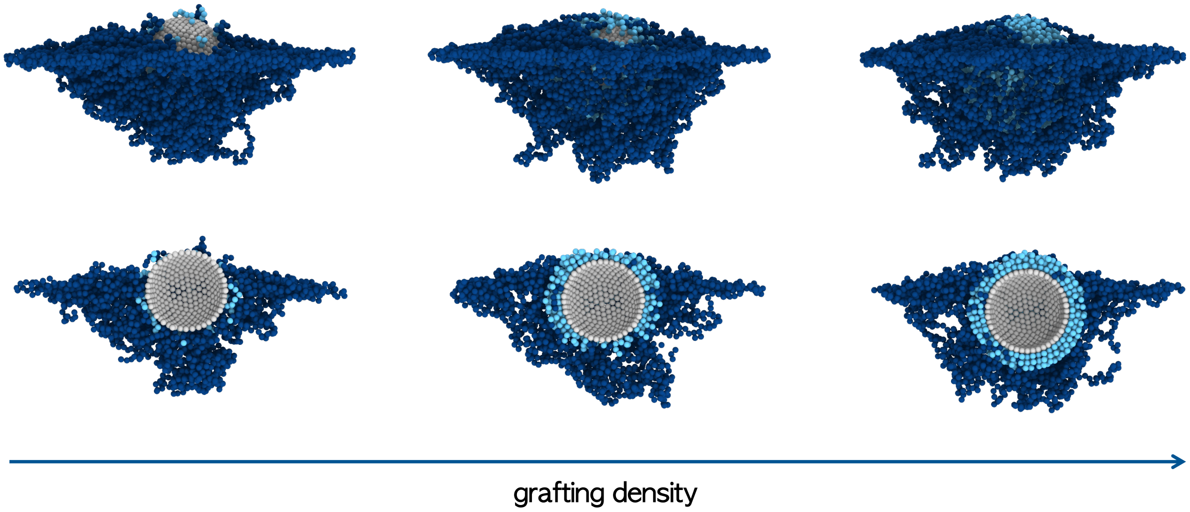

Figure S7: (Top) Full and (bottom) cross-section simulation snapshots showing core-shell microgels adsorbed at an interface for increasing grafting density at the same shell thickness. Specifically, CS_m^{10}, CS_m and CS_m^{100} are shown (from left to right). Blue beads belong to the shell of the microgel, light blue is for the added polymer chains through which the grafting density is tuned, and grey is for core beads.
